# Enhancing stock index prediction: A hybrid LSTM-PSO model for improved forecasting accuracy

**DOI:** 10.1371/journal.pone.0310296

**Published:** 2025-01-14

**Authors:** Xiaohua Zeng, Changzhou Liang, Qian Yang, Fei Wang, Jieping Cai

**Affiliations:** School of Economics and Trade, Guangzhou Xinhua University, Dongguan, China; NUST: National University of Sciences and Technology, PAKISTAN

## Abstract

Stock price prediction is a challenging research domain. The long short-term memory neural network (LSTM) widely employed in stock price prediction due to its ability to address long-term dependence and transmission of historical time signals in time series data. However, manual tuning of LSTM parameters significantly impacts model performance. PSO-LSTM model leveraging PSO’s efficient swarm intelligence and strong optimization capabilities is proposed in this article. The experimental results on six global stock indices demonstrate that PSO-LSTM effectively fits real data, achieving high prediction accuracy. Moreover, increasing PSO iterations lead to gradual loss reduction, which indicates PSO-LSTM’s good convergence. Comparative analysis with seven other machine learning algorithms confirms the superior performance of PSO-LSTM. Furthermore, the impact of different retrospective periods on prediction accuracy and finding consistent results across varying time spans are. Conducted in the experiments.

## 1. Introduction

The development of electronic trading systems and computing technology have made stock trading more efficient since the end of the 20th century. The stock market has experienced tremendous expansion and generated a large amount of stock trading data and information. Therefore, how to grasp the operating mechanism of stock price movements has become a hot topic for researchers. However, the price trend in the stock market is a complex nonlinear dynamic system [1]. The time series of the stock price are influenced by the internal micro environment of enterprises, such as company performance and growth potential. In addition, stock prices are also influenced by external macroeconomic factors, such as changes in Gross Domestic Product(GDP), market interest rates, and media opinion etc. [2]. The fluctuation of stock prices is described as a stochastic process [3]. Econometric models are used to describe stock behavior, such as traditional time series prediction methods, exponential smoothing, and differential integrated moving average autoregressive models etc. [4, 5].

In recent years, artificial intelligence technology has been widely used to solve complex nonlinear time series stock prediction problems [6]. Machine learning and deep learning are the main effective methods to predict stock prices. By constructing neural networks, the models have the ability to simulate the human brain to analyze, learn, and interpret data such as images, sound, and text. Among them, a common model is to solve stock price prediction as a regression problem of time series. The common regression metrics used to measure the prediction performance included mean square error (MSE), root mean square error (RMSE), mean absolute error (MAE), mean absolute percentage error (MAPE), and goodness of fit (R^2^) of the model [7–10].

The methods proposed in many studies improve the performance of LSTM models by in many aspects, there are still some potential limitations and sources of error or bias. (1) computational complexity. The combination with optimization algorithms such as adaptive genetic algorithms may increase the computational complexity, resulting in longer training time. (2) Model generalization ability. Although hyperparameters are optimized, the generalization ability of the model under different market conditions (such as bull and bear markets) may be limited, requiring more validation and testing. (3) The influence of randomness. Meta-heuristic algorithms have randomness, which need to be run multiple times to ensure the stability of the results. (4) Data quality and quantification strategies. Data quality and pre-processing methods can handle noise and outliers of stock price, which have an important impact on prediction results. Particle Swarm Optimization (PSO) is a swarm intelligence algorithm that has unique advantages due to its high efficiency.

In this study, a novel PSO-LSTM stock price prediction model is proposed to leverage PSO in order to optimize LSTM hyperparameters. The decision to employ PSO for hyperparameter optimization stems from its efficacy in addressing complex optimization problems, particularly in high-dimensional spaces encountered in deep learning models like LSTMs. PSO excels in searching for global optima, converging relatively quickly compared to other optimization techniques, thus efficiently exploring hyperparameter combinations and saving computational resources. Moreover, the non-convex nature of hyperparameter optimization in LSTMs necessitates robust algorithms like PSO, capable of navigating complex, non-linear search spaces. Additionally, PSO’s ease of implementation and tuning make it accessible for researchers and practitioners seeking effective hyperparameter optimization strategies. By leveraging PSO, the predictive performance of LSTM-based stock price prediction model will be enhanced while addressing the challenges posed by high-dimensional optimization tasks. In addition, stock prices are high noise and high-dimensional time series, data preprocessing methods and feature selection strategies are also important for improving the performance of stock prediction models. Therefore, the model proposed in this study consists of three parts: (1) Data preprocessing includes wavelet transform (WT) and correlation analysis. WT was applied for the denoising time series. Correlation analysis is used to select the characteristics of input variables. (2) PSO is used to optimized the number of optimization iterations and the number of hidden neurons in LSTM neural networks. (3) The hyperparameters of the optimal solution obtained from PSO optimization are used as inputs of the LSTM model to predict stock prices. By comparing LSTM models with different hidden layers, the PSO-LSTM stock prediction results with the best performance will be selected as outputs. In order to analyze and compare the predicted results, RMSE, MAE, MAPE, and R^2^ are applied to evaluate the regression models.

The efficient market hypothesis (EMH) assumes that the degree of market development is heterogeneous. Selecting indices from different levels of market development can help explain the robustness of algorithms as market conditions may potentially affect the effectiveness of stock forecasting. Therefore, six stock indices are used to test the performance of the model. These indices include the Dow Jones Industrial Average (DJIA) index of the New York Stock Exchange, Standard & Poor’s 500 (S&P 500) index, Nikkei 225 index of Tokyo, Hang Seng Index of Hong Kong market, CSI300 index of Chinese Mainland stock market, and Nifty50 index of India. Among these six stock indices, DJIA and S&P 500 represent the most developed and efficient market. Hang Seng Index and Nikkei 225 represent the middle stage between efficient and inefficient markets. CSI300 and Nifty50 represent developing markets.

The main contribution and novelty of this article are as follows. Comprehensive experimental analysis utilizing data from six global stock indices across markets of varying development levels can facilitate a thorough comparative study of model performance. Then, the introduction of a novel PSO-LSTM stock price prediction model can leverage particle warm optimization to optimize LSTM hyperparameters. Finally, Investigation into the influence of diverse retrospective periods (50 days, 20 days, and 7 days) on model performance, providing insights into the model’s efficacy across different prediction cycles for practical applications.

The remainder of this article is organized as follows. Section 2 mentions the related work. Section 3 introduces the methodology applied in this study. Section 4 presents the details of the experimental design. Section 5 illustrates the experimental results. Section 6 summarizes the research.

## 2. Related work

Machine Learning (ML) has emerged as a transformative tool across various scientific and industrial domains. In the realm of environmental science, ML has been applied to landslide susceptibility mapping (LSM) in the Three Gorges Reservoir area. Utilizing Automated Machine Learning (AutoML), this approach simplifies the modeling process for non-experts by automating the selection and tuning of models. The implementation of AutoML has shown a significant performance improvement. ML’s utility is also evident in geotechnical engineering, particularly in predicting reservoir landslide displacements [11]. An Earthworm Optimization Algorithm-optimized Support Vector Regression (EOA-SVR) model has been developed, surpassing traditional metaheuristic models in stability and performance. This method provides a reliable tool for medium and long-term landslide early warning systems, which is crucial for disaster management and mitigation. As for post-disaster analysis, ML techniques, combined with multitemporal remote sensing, were used to monitor and analyze the evolution of landslide activity over a decade [12]. The use of algorithms like Random Forest facilitated the accurate mapping of landslides, revealing a gradual decrease in occurrences over time, despite intermittent spikes due to monsoonal rains. The study of long-term hydrological changes in the pan-Arctic region illustrates ML’s role in environmental monitoring [13]. By employing algorithms like the Extreme Gradient Boosting Tree (XGBoost), researchers have reconstructed historical water levels of pan-Arctic lakes, integrating these findings with climatic and hydrological data. This application not only enhances the understanding of hydrological dynamics influenced by climate change but also provides a methodological blueprint for similar environmental studies. These instances underline ML’s broad applicability and potential in providing innovative solutions to complex problems across various disciplines, reinforcing its role as a cornerstone technology in modern science and engineering [14].

Deep learning (DL) is a sub field of ML. LSTM neural network proposed by Hochreiter and Schmidhuber has shown excellent performance in time series prediction [15]. Compared to Convolutional Neural Network (CNN), LSTM neural network improves gate structure to selectively learn useful hidden information from a large amount of complex historical data. Therefore, LSTM can better understand the patterns and trends of time series [16]. Reference [17] used LSTM to predict the returns on investment portfolios of the S&P 500 index. The experimental results show that LSTM performs better than Random Forest (RF), Deep Neural Network (DNN), and Logistic Regression (LR). Reference [18] used LSTM to predict the opening prices of Google and NKE stocks and the results demonstrated that the LSTM model had good predictive ability. Reference [19] established an LSTM prediction model, using historical prices and technical analysis indicators as input variables to predict future trends in stock prices. LSTM was compared with other machine learning methods, and the experimental results showed that LSTM has excellent performance. Reference [20] compared LSTM with Support Vector Regression (SVR) using 9 common technical indicators to predict 5 US stock prices of international companies. The results showed that LSTM had better average prediction accuracy than SVR.

The accuracy and convergence of LSTM highly depend on the combination of hyperparameters, such as the number of hidden layers and neurons in each layer. However, the network topology constructed by these hyperparameters are difficult to manually adjust one by one, which makes it difficult to ensure a suitable and optimal network structure that meets practical applications. Metaheuristic algorithm is an optimization technique that can be used to optimize the hyperparameters of machine learning models [21, 22]. Compared with traditional optimization methods which may fall into local minima and fail to find the optimal solution, metaheuristic algorithms can find the optimal hyperparameters even under conditions of complex data distribution and huge search space [23–25].

PSO [26] is a swarm intelligence algorithm that has unique advantages among metaheuristic algorithms due to its high efficiency. The principle of PSO algorithm is to simulate the process of birds searching for food, dynamically adjusting their position based on individual and group extremum. PSO searches for the best solution to a given problem in the search space using a group of candidate solutions called particles. The iterative process of the algorithm only has a small number of parameters that need to be adjusted, such as particle velocity, particle quantity, and particle position. Based on the best position of each particle in the search space and the best position of the population so far, the position and velocity of the particles will be changed during each iteration. As particles move towards the best place, unknown domains in the search space are automatically avoided. This iterative optimization help particles to avoid local minima and converge to the global optimal solution. Moreover, the adaptability of PSO algorithm enables LSTM to determine the optimal parameters based on data features quickly and accurately, which can also help reduce the computational cost of adjusting the model. The combination of adaptive PSO algorithm and self-learning iterative optimization of key parameters of LSTM model can avoid manual parameter adjustment and improve the efficiency and authenticity of the model [27, 28].

Many types of LSTM (and other RNNs) have been tuned by metaheuristics algorithms for various purposes. Parkinson’s disease diagnosis is a challenging task due to the absence of reliable tests. Cuk et al. explored the potential of LSTM neural networks combined with attention mechanisms and proposed an optimized crayfish algorithm to detect Parkinson’s disease accurately [29]. The method achieved promising results with an accuracy of 87.42% using dual-task walking test data. Quick process shift detection is vital for modern smart manufacturing. Yang et al. proposed single and stacked LSTM models optimized with metaheuristic optimizers to detect shifts in high-dimensional manufacturing processes [30]. The CSOS_S_LSTM model achieved the best results with a shorter out-of-control run length, improving response time by 38.77% on average. Bacanin et al. proposed the use of a long short-term memory (LSTM) deep learning model for cloud load time-series forecasting [31]. It utilized LSTM with attention layers and a modified particle swarm optimization (PSO) algorithm. The variational mode decomposition (VMD) was used for data preprocessing. The proposed methodology outperformed other techniques in terms of performance metrics. SHAP analysis was used to assess feature importance. The methodology had potential for assisting cloud providers in resource allocation and provisioning decision-making processes. Francisco J. employed the Automated Machine Learning (AutoML) process for feature selection, model creation, and hyperparameter optimization to develop a machine learning model for Google stock price forecasting [32]. The AutoML process selected features from 11 technical indicators. Hyperparameters were optimized using PSO, achieving accurate results with errors ranging from 1E-2 to 9E-4. The CNN-LSTM network outperformed the standalone LSTM model. Pedroza-Castro et al. addressed the need for efficient computation resource management in distributed cloud-based services by proposing a methodology for forecasting cloud resource load using RNN with attention layers [33]. The models were optimized through hyperparameter tuning using a modified PSO metaheuristic and incorporated variational mode decomposition for handling non-stationary data sequences. The study demonstrated the potential of the proposed method in accurately forecasting cloud load. The results outperformed state-of-the-art algorithms and provided valuable insights for cloud providers. To enhance the security of intrusion detection systems, Donkol introduced an enhanced LSTM technique integrated with RNN (ELSTM-RNN) [34]. The proposed system employed PSO to select effective features and enhanced LSTM for classification. It addressed the challenges faced by existing methods and achieved better performance in detecting intrusions within network communications. The system’s efficiency was validated through extensive testing on multiple datasets, demonstrating improved classification accuracy and faster training times compared to existing methods.

In the latest research, many studies have applied the PSO algorithm to the field of time series prediction. The combination of PSO and LSTM models is used to predict the trend of stock changes based on quantitative and textual information [35]. Empirical results showed that the model was superior to the BP neural network and LSTM network models. Reference [36] validated the effectiveness and applicability of the PSO-LSTM model based on stock prices. Reference [37] validated the effectiveness of the PSO-LSTM model based on the sales of five types of fishing gear in an online store and two publicly available datasets.

Therefore, the proposed PSO-LSTM hybrid model for stock price prediction is deliberate and multifaceted. PSO’s compatibility with LSTM architecture, coupled with its proven track record and efficiency in optimization tasks, makes it a suitable choice for stock price prediction. Furthermore, PSO aligns optimally with the requirements and characteristics of stock price prediction due to its ability to effectively handle the complex and dynamic nature of stock market data. The inherent swarm intelligence of PSO allows it to navigate the intricate parameter space of LSTM, facilitating the discovery of optimal solutions amidst the inherent noise and non-linearity of financial markets. Additionally, the adaptability of PSO enables it to continuously refine its search strategy, ensuring robust performance in the face of evolving market conditions. PSO is also underpinned by the No Free Lunch theorem [38], which underscores the recognition that while other optimization algorithms may excel in certain contexts. PSO offers the most suitable optimization approach for stock price prediction framework.

## 3. Methodology

### 3.1 Long short-term memory neural networks

LSTM has been widely applied in predicting stock prices in recent years. Traditional RNN is prone to the problem of vanishing gradients. By introducing forget gates and memory units, LSTM can solve the above problems. Due to the presence of memory units, the flow of information is achieved through a mechanism called cellular state. LSTM can selectively retain or forget information based on its importance, which achieves dynamic learning of data patterns and improves prediction accuracy. LSTM can also overcome the long-term dependency problem in recurrent networks as gradients can flow over a longer period of due to the introduction of self recurrent paths.

**Fig 1:**
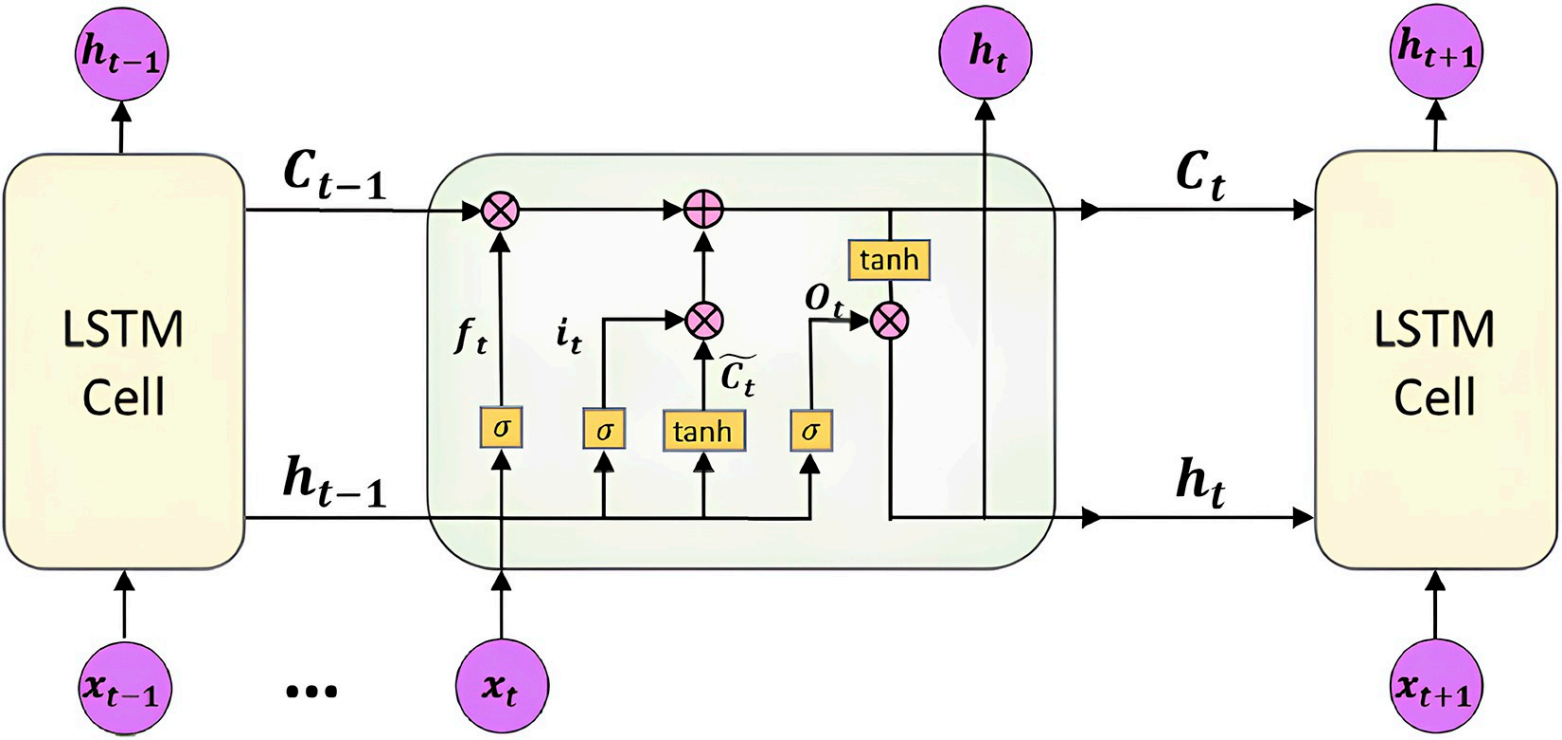
LSTM unit.

In addition, the original model improvement of LSTM also enhances the reliability of the network [39]. This improvement stems from the structure of the LSTM unit. LSTM includes a unit specifically designed for long-term storage of information. LSTM also consist of input gate, output gate, and forget gate for precise control of data flow. During the training process, the LSTM neural network adopted the Time Series Back propagation (BPTT) algorithm [40], which further improved the performance of the model. The internal structure of the LSTM computing unit is shown in Fig 1.

The gate control operations consist of a sigmoid activation function and a dot product operation. The forget gate, input gate, and output gate are the three gates used by LSTM to protect and control the state of a single computing unit.

The forgetting gate determines the information that needs to be discarded, which can be expressed as Eq (1).

$$f_{t}=\sigma (w_{f}*[h_{t-1},x_{t}]+b_{f})$$ (1)

Where *w*_*f*_ represents the connection weight of the previous output. The symbol h_*t*−1_ is the previous output. The symbol *x*_*t*_ is the current input. The symbol is the bias vector. The symbol *σ* is an activation function.

The input gate determines the information that needs to be updated. It is obtained by multiplying two vectors created by the input gate layer and tanh layer that expressed in Eqs (2) and (3).


$$i_{t}=\sigma (W_{i}∙[h_{t-1},x_{t}]+b_{i})$$ (2)

$${\tilde{C}}_{t}=tanh(W_{C}∙[h_{t-1},x_{t}]+b_{C})$$ (3)

The output gate updates the information of the input gate and the forget gate by Eqs (4) and (5). First, the sigmoid activation function determines the output part. Then the cell state is normalized to [–1,1] through the tanh layer. Finally, dot multiplication is performed.


$$C_{t}=f_{t}*C_{t-1}+i_{t}*{\tilde{C}}_{t}$$ (4)

$$o_{t}=\sigma (W_{o}[h_{t-1},x_{t}]+b_{0})$$ (5)

Then the final output value of cell is calculated by Formula (6).


$$h_{t}=o_{t}*tanh(C_{t})$$ (6)

**Fig 2:**
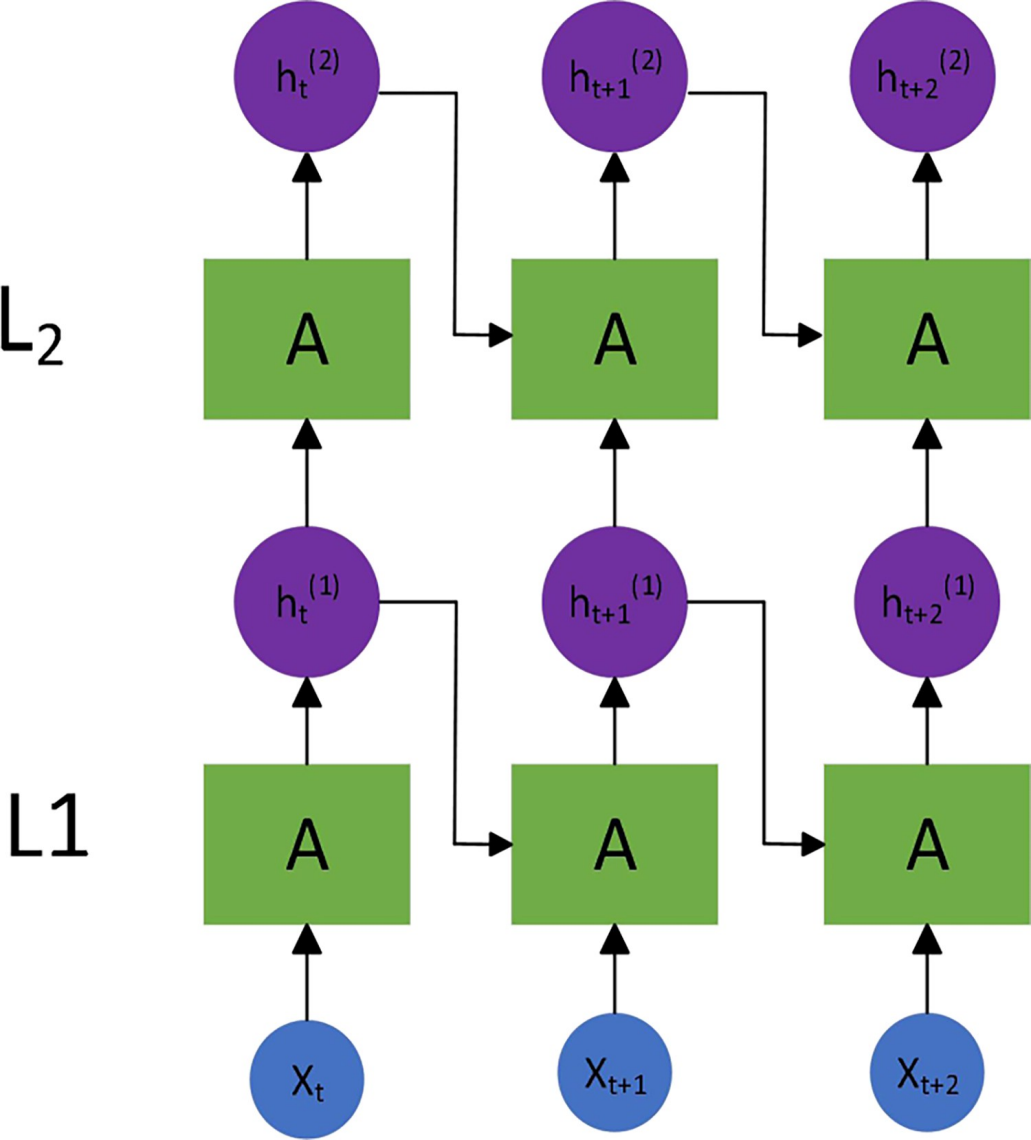
Typical LSTM neural network.

The LSTM neural network can be obtained by connecting each LSTM unit with a directed graph structure. Fig 2 shows a typical network structure.

The training method of LSTM model usually include two steps. First, the output value of LSTM units is calculated through forward propagation. Then back propagation is used to calculate the error value and the weight gradient. These gradient values are used for updating the calculus gradient descent algorithm. Most neural network optimization algorithms use stochastic gradient descent (SGD), adaptive gradient algorithm (AdaGrad), and adaptive moment estimation algorithm (Adam). The SGD algorithm maintains a single learning rate during execution. Adam function is used in this article which can simultaneously calculate first-order moment estimation and second-order moment estimation. The calculation enable each parameter to obtain independent adaptive learning rates, thereby reducing overfitting problem to a certain extent [41]. In the specific experimental process, hyperparameters such as batch size, number of hidden layer neurons, and number of hidden layers for LSTM are needed to be set by the experimental designer.

### 3.2 Particle swarm optimization (PSO) algorithm

PSO algorithm will be used to optimize the hyperparameters of LSTM neural networks in this study. The core of PSO optimization algorithm is based on cooperation and information sharing among particles in the population. The optimal solution is obtained through iteration. For instance, there are N particles in the D-dimensional search space. The position and velocity of each particle are random during initialization. The current optimal extremum for each particle is the optimal solution obtained by the current individual search (particle best, *pbest*). The expression of the optimal solution of *i*^*th*^ particle is $pbset_{i}=(p_{i1},p_{i2},\ldots ,p_{iD})$. The expression of the global extremum is $gbest=(p_{g1},p_{g2},\ldots ,p_{gD})$. All particles in the particle swarm will update their velocity and position according to Eqs (7) and (8) until the optimal solution is found [39].


$$\nu_{id}^{k}=wv_{id}^{k-1}+c_{1}r_{1}(pbest_{id}-x_{id}^{k-1})+c_{2}r_{2}(gbest_{d}-x_{id}^{k-1})$$ (7)

$$x_{id}^{k}=x_{id}^{k-1}+v_{id}^{k-1}$$ (8)

The symbol *k* represents the number of iterations. The spatial position of *i*^*th*^ particle is $x_{i}=(x_{i1},x_{i2},\ldots ,x_{id})$. The speed of *i*^*th*^ particle is $v_{i}=(v_{i1},v_{i2},\ldots ,v_{id})$. The symbol *w* is the inertia factor used to adjust the search range of the solution space which represents the tendency of particles to maintain their historical velocity. The symbol *c*_1_ and *c*_2_ is an acceleration constant used to adjust the maximum learning step size. The symbol *r*_1_ and *r*_2_ is a uniform random number used to increase the randomness of the search, with a range of values between [0, 1]. In addition to the inertia of particles, Eqs (7) and (8) also consider the following two tendencies. One is the tendency of particles that approach their historical best position. The other is the tendency of particles that approach the historical best position of a population or neighborhood.

Subsequently, the fitness value of each particle is evaluated based on Eqs (9) and (10). The current fitness value of each particle will be compared and ranked with the fitness value of the global best position (*gbest*) [39]. The *pbest* with better fitness values will be updated based on the new global best position *gbest*. If the number of iterations of the algorithm reaches its maximum value, the extreme value of the particle swarm is taken as the optimal solution. Otherwise, the particle swarm will continuously iterate the above process until the optimal solution is found.

$$pbest_{i}(k)=\{\begin{array}{l} pbest_{i}(k-1),\;if\;f(x_{i}(k))\geq pbest_{i}(k-1)\\ \;x_{i}(k),\;other \end{array}$$ (9)

$$gbest_{i}(k)=min\{f(pbest_{i}(k))\}$$ (10)

Algorithm 1 PSO Algorithm

procedure **PSO**

 for each particle i

  Initialize velocity Vi and position Xi for particle /

  Evaluate particle i and set Pbesti = Xi

 end for

 Gbest = min {Pbesti}

 while not stop

  for i = 1 to N

  Update the velocity and position of particle

  Evaluate particle

  if fit (Xi) < fit (Pbesti)

   Pbesti = Xi,

  if fit(Pbesti) < fit (Gbest)

   Gbest = Pbest,

  end for

 end while

 print Gbest

end procedure

### 3.3 PSO-LSTM stock price prediction model

**Fig 3:**
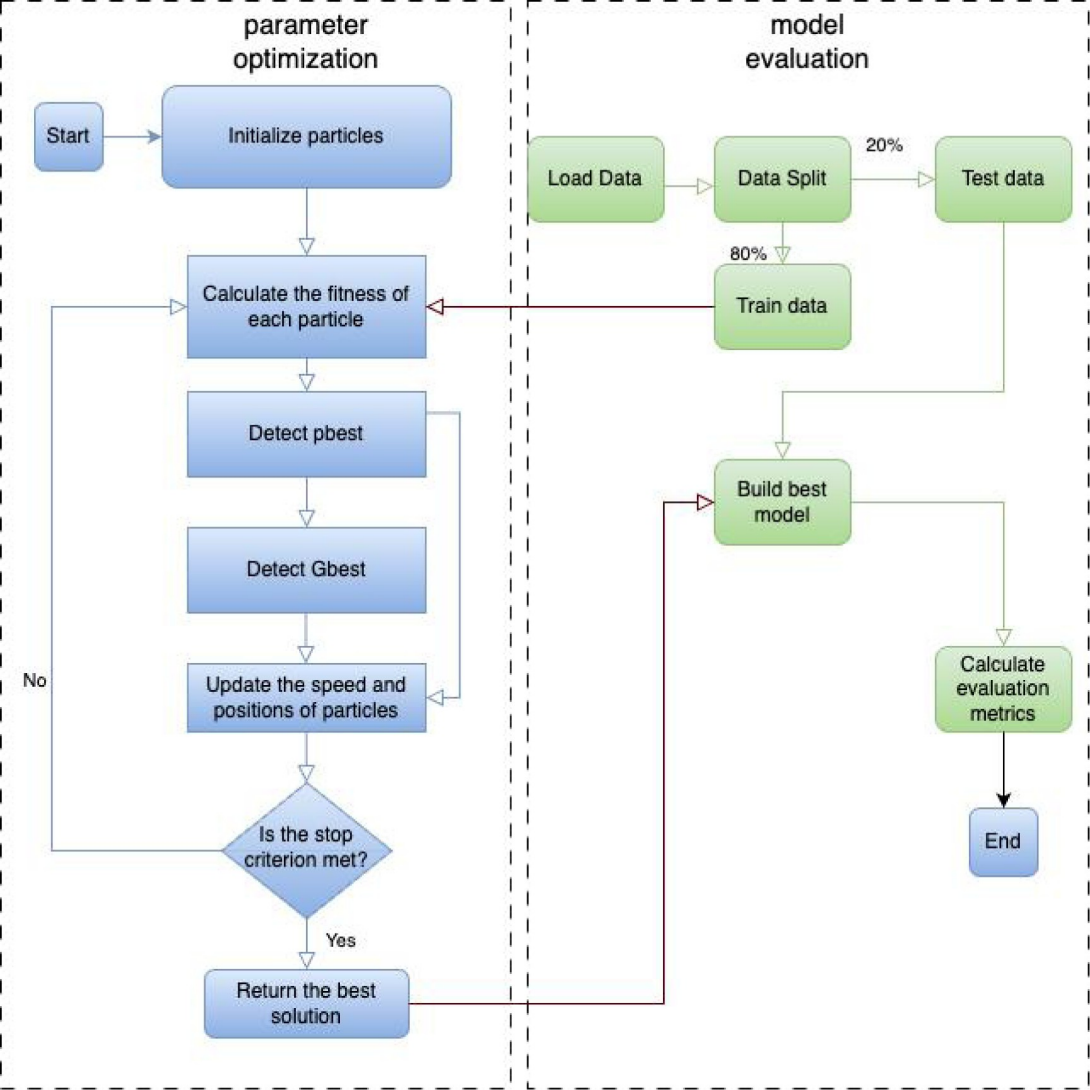
The flowchart of the PSO-LSTM stock price prediction model.

The number of optimization iterations and the number of neurons in the hidden layer are used as particles. The flowchart of the PSO-LSTM stock price prediction model proposed in this article is shown in Fig 3.

Three LSTM neural networks with one hidden layer, two hidden layers, and three hidden layers will be constructed. The number of iterations and the number of neurons in different hidden layers are used as the optimization objectives of the model. This experiment will preprocess six stock index datasets. At the same time, the dataset will be divided into training and testing sets. The root mean square error (RMSE) of the prediction result is used as the result of the fitness function value. *pbest* and *gbest* will be updated according to Algorithm 1 and the optimal solution will be selected as outputs. The hyperparameters of the optimal solution are input into the LSTM model to predict stock prices. The results of PSO-LSTM stock prediction models with different hidden layers will be compared based on the evaluation indicators.

## 4 Research data and experiment

The six stock indices used to test the performance of the model include the Dow Jones Industrial Average (DJIA) index of the New York Stock Exchange, Standard & Poor’s 500 (S&P 500) index, Nikkei 225 index of Tokyo, Hang Seng Index of Hong Kong market, CSI300 index of Chinese Mainland stock market, and Nifty50 index of India. The data are from the WIND database (http://www.wind.com.cn) provided by Shanghai Wind Information Co., Ltd, CSMAR database (http://www.gtarsc.com) provided by Shenzhen GTA Education Tech. Ltd., and the global financial portal Investing.com. The time span were from 2008/07/02 to 2016/09/30.

### 4.1 Input variables

Stock prices are influenced by both macro and micro environments. Therefore, three sets of variables are selected as input variables which are shown in Table 1. The first set of input variables are the historical trading data which include open, high, low, close prices and the trading volume [42]. These raw prices represent fundamental trading information. The details are described as No.1-5 in Table 1. The second set of input variables consists of 12 technical indicators that can grasp moving trends of stock price [43]. The details are described as No.6-15 in Table 1. The final set of inputs is the macroeconomic indicators, which include the exchange rate and interest rate [44].

**Table 1:** Considered features and their types.

No.	Feature name	Explaining	Type	In/Out
**1**	Open Price	nominal daily open price	Numerical	Input
**2**	Close Price	nominal daily close price	Numerical	Input
**3**	Low Price	nominal daily highest price	Numerical	Input
**4**	High Price	nominal daily lowest price	Numerical	Input
**5**	Trading volume	Daily trading volume	Numerical	Input
**6**	MACD	Moving average convergence divergence: displays trend following characteristics and	Numerical	Input
**7**	CCI	Commodity channel index: helps to find the start and the end of a trend.	Numerical	Input
**8**	ATR	Average true range: measures the volatility of price.	Numerical	Input
**9**	BOLL	Bollinger Band: provides a relative definition of high and low, which aids in rigorous pattern recognition	Numerical	Input
**10**	EMA20	20 day Exponential Moving Average	Numerical	Input
**11**	MA5/MA10	5/10 day Moving Average	Numerical	Input
**12**	MTM6/MTM12	6/12 month Momentum: helps pinpoint the end of a decline or advance	Numerical	Input
**13**	ROC	Price rate of change: shows the speed at which a stock’s price is changing	Numerical	Input
**14**	SMI	Stochastic Momentum Index: shows where the close price is relative to the midpoint of the same range.	Numerical	Input
**15**	WVAD	Williams’s Variable Accumulation/ Distribution: measures the buying and selling pressure.	Numerical	Input
**16**	Exchange rate	US dollar Index	Numerical	Input
**17**	Interest rate	Interbank Offered Rate	Numerical	Input
**18**	Predicted Close Price	Close Price in next day	Numerical	Output

### 4.2 Correlation analysis

From the perspective of stock trading, the closing price is an important factor in formulating trading strategies. To avoid multicollinearity, the numerical values of Pearson correlation coefficients between the closing price and other features are calculated. Table 2 show the results of Pearson correlation coefficients of DJIA as an sample. SPSS analysis shows that features with correlation coefficients above 95% have a significant impact on price fluctuations (** Correlation is significant at the 0.01 level (2-dailed)). Therefore, features with higher correlation coefficients will be removed.

**Table 2:** Pearson correlation coefficient of DJIA.

	Open	High	Low	Close	Volume	MACD	CCI	ATR	BOLL	EMA20	MA10	MTM6	MA5	MTM12	ROC	SMI	WVAD	US DOLLAR INDEX	FEDERAL FUND
Pearson	1.000	1.000	1.000	1.000	-0.770	0.196	0.011	-0.150	0.994	0.997	0.998	0.058	0.999	0.107	0.075	0.072	0.054	0.617	-0.130
Sig.(2-tailed)	0.000 **	0.000 **	0.000 **	0.000 **	0.000 **	0.000 **	0.603	0.000 **	0.000 **	0.000 **	0.000 **	0.008	0.000 **	0.000**	0.001**	0.001**	0.014	0.000 **	0.000 **

** Correlation is significant at the 0.01 level (2-dailed).

**Fig 4:**
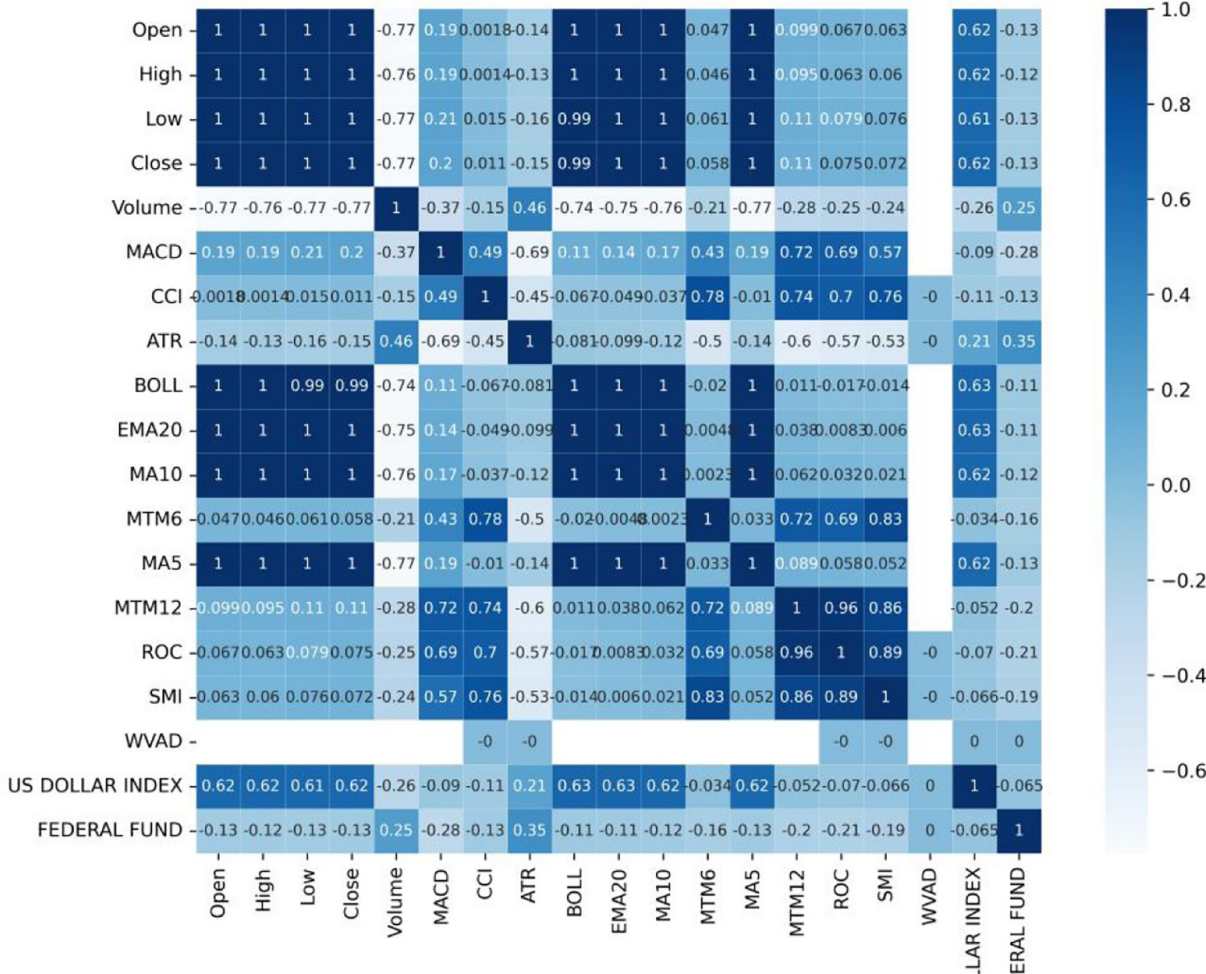
DJIA correlation heatmap.

To further demonstrate the relevance, correlation heatmap of DJIA is illustrated as Fig 4.

### 4.3 Data pre-processing

#### 4.3.1 Data denoising

Due to the complexity and high-noise of the stock market, data denoising is a necessary means to improve the performance of LSTM models. Wavelet transform (WT) has the ability to simultaneously analyze the frequency components of financial time series. Therefore, WT can process highly non-stationary financial time series data [45]. The Haar function is used as the wavelet function in the study, which not only decomposes time series into time-domain and frequency-domain, but also has the advantage of short computation time [46].

**Fig 5:**
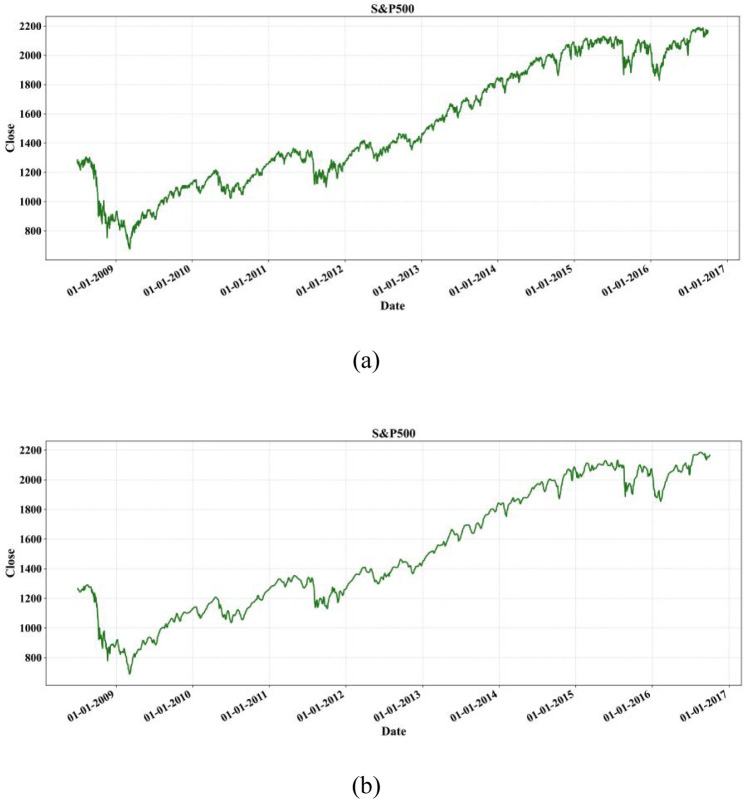
Comparison of closing price of the S&P500 index before denoising (a) and after denoising (b).

Continuous Wavelet Transform (CWT) extracts features of time series in the dimensions of time and scale, but the coefficients contain a large amount of redundant information and require further dimensionality reduction. Therefore, Discrete Wavelet Transform (DWT) has gradually become a more common method, which can extracts features more effectively by decomposing time series into orthogonal component sets. Fig 5 expressed the closing price of the S&P500 index for each trading day from July 1, 2008 to October 1, 2016. Fig 5(A) shows the original historical closing prices without noise reduction, which are unstable and noisy. The Pywt library in Python was used for DWT. Fig 5(B) shows the closing price of the S&P500 index after three-layer wavelet decomposition, with a more stable sequence and less noise.

#### 4.3.2 Data normalization

There are three sets of input variables with different dimensions used in the experiments. Therefore, it is necessary to standardize the data to be within the output range of the activation function. MinMaxScaler [47] in Pandas is used to process different scaled features into the ranges [–1,1] based on Eq (11). Specifically, as data normalization preserves all relationships in the data precisely, it avoids bias [48].

$$x_{norm}=\frac{x-x_{min}}{x_{max}-x_{min}},$$ (11)

where, *x*_*norm*_ is the converted value. *x*_*max*_ is the maximum value of the sample and *x*_*min*_ is the minimum value of the sample.

### 4.4 Experimental Environment and evaluation indicators

The deep learning framework TensorFlow in the Keras framework [49] is served as back end support to construct prediction models. Tables 3–5 provide the details of the experimental environments.

**Table 3:** Experimental environment of hardware.

CPU	Intel(R) Intel Xeon E3-1230v6@3.50GH CPU
GPU	NVDIA Quadro M2000
RAM	16GB

**Table 4:** Experimental environment of software.

Operating System	Windows 10
Software platform Framework	Jupyter Notebook Keras

**Table 5:** The parameters of LSTM training model.

Name of parameters	Value/Method
Number of input layer node	50
Number of output layer node	1
Activation function	ReLU
Regularization method	Dropout
Training method	Adam

Six stock indices were used to train and test predictive models in this experiment. The closing price is used as the predicted value, and the difference between the predicted value and the true value is used as the predictive effect. The evaluation indicators used in this study which are shown as Eqs (12)–(15) were root mean square error (RMSE), mean absolute error (MAE), mean absolute percentage error (MAPE), and goodness of fit (R^2^) of the model [7–10]. RMSE is defined as

$$RMSE=\sqrt{\frac{1}{n}\sum_{i=1}^{n}{\left(y_{i}-{\hat{y}}_{i}\right)}^{2}},$$ (12)

MAE is given as

$$MAE=\frac{\sum_{i=1}^{n}\left|y_{i}-{\hat{y}}_{i}\right|}{n},$$ (13)

MAPE is defined as

$$MAPE=\frac{\sum_{i=1}^{n}\left|(y_{i}-{\hat{y}}_{i})/y_{i}\right|}{n}\times 100,$$ (14)

R^2^ is defined as

$$R^{2}=1-\frac{\sum_{i=1}^{n}{\left(y_{i}-{\hat{y}}_{i}\right)}^{2}}{\sum_{i=1}^{n}{\left(y_{i}-\bar{y}\right)}^{2}}.$$ (15)

The RMSE, MAE, and MAPE were used to measure the deviation between the actual and predicted values. The smaller the value, the closer the predicted value to the actual value. R^2^ was used to measure the degree of model fitting. The closer it was to 1, the better the model fits the actual prices.

## 5. Experimental results of PSO-LSTM forecasting model

### 5.1 Experiments of PSO-LSTM model (Parameters manually set)

Firstly, the results of PSO optimized LSTM model with one hidden layer, two hidden layers, and three hidden layers will evaluated in this experiment. The parameters for initializing PSO will be based on Eqs (7) and (8). The group size is set to 20. The range of the number of neurons and iteration times in the hidden layer are set to [0, 300]. The range of particles is set to [0, 300]. The speed range of particles is set to [–2, 2]. The maximum number of iterations for PSO is set to 50. The inertia weight *w* is a major parameter. The larger the inertia weight *w* is, the stronger the global optimization ability is and the weaker the local optimization ability is. *w* is set to 0.8 in the experiment. The acceleration constants *c*_1_ and *c*_2_ are set to 1.5, indicating that the weights for individual and population particles are the same. The first 80% of the data are used as the training set while the last 20% are used as the testing set. For each experiment, 10 tests are conducted and evaluated using the average of the results. In section 4.1, the impact of manually setting the number of neurons on the experimental results is discussed. Due to too many combinations, a set of experiments are selected to present the results. In Section 5.1 and 5.2, a 50 day retrospective period is used to validate the model.

### 5.1.1 Impact of increasing the number of hidden neurons on experimental results

The prediction errors of different hidden neurons when a hidden layer is used and training rounds are set to 100 is shown in Table 6. It can be seen that increasing the numbers of hidden neurons has no significant effect on the experimental results.

**Table 6:** The prediction errors of different hidden neurons. (Hidden layer = 1, epochs = 100).

Index	Hidden layers	Hidden neurons	Epochs	RMSE	MAE	MAPE	R2
DJIA	1	50	100	146.057	111.664	0.006	0.947
	1	100	100	125.238	88.608	0.005	0.961
	1	150	100	**120.476**	**85.788**	**0.005**	**0.964**
	1	200	100	133.657	95.981	0.006	0.955
	1	250	100	124.222	83.182	0.005	0.961
	1	300	100	150.589	117.661	0.007	0.943
S&P500	1	50	100	24.447	21.591	0.010	0.882
	1	100	100	21.119	16.169	0.008	0.912
	1	150	100	25.828	23.097	0.011	0.868
	1	200	100	**14.523**	**10.379**	**0.005**	**0.958**
	1	250	100	15.101	10.437	0.005	0.955
	1	300	100	27.149	24.422	0.012	0.854
HangSeng	1	50	100	227.189	146.647	0.006	0.991
	1	100	100	219.629	144.966	0.006	0.992
	1	150	100	202.181	128.335	0.006	0.993
	1	200	100	215.227	144.319	0.006	0.992
	1	250	100	**199.881**	**141.105**	**0.006**	**0.993**
	1	300	100	202.211	131.297	0.006	0.993
Nikkei225	1	50	100	**312.286**	**239.183**	**0.013**	**0.961**
	1	100	100	393.880	282.991	0.016	0.937
	1	150	100	550.485	447.644	0.024	0.878
	1	200	100	406.219	334.019	0.018	0.933
	1	250	100	1469.100	1125.776	0.061	0.130
	1	300	100	365.941	264.629	0.015	0.946
CSI300	1	50	100	128.263	59.518	0.016	0.951
	1	100	100	108.647	55.285	0.015	0.964
	1	150	100	117.986	61.657	0.017	0.958
	1	200	100	108.358	48.077	0.013	0.965
	1	250	100	112.065	52.160	0.014	0.962
	1	300	100	**106.960**	**53.733**	**0.014**	**0.966**
Nifty50	1	50	100	69.764	53.587	0.007	0.976
	1	100	100	94.991	76.457	0.009	0.955
	1	150	100	70.463	54.266	0.007	0.975
	1	200	100	64.743	50.256	0.006	0.979
	1	250	100	62.110	47.164	0.006	0.981
	1	300	100	**56.198**	**41.258**	**0.005**	**0.984**

#### 5.1.2 The impact of different training rounds on experimental results

Next, the impact of different training rounds in the LSTM model is considered. The prediction errors for different training rounds when the number of hidden neurons and hidden layers are set to 200 and 1 respectively are shown in Table 7. As the number of training rounds increases, the model can better extract features from the data set, which reduce the prediction error of the model. The first number of hidden neurons represents the number of neurons in the first hidden layer of the LSTM model, the second number of hidden neurons represents the number of neurons in the second hidden layer, and so on. However, if the number of training rounds increases beyond the appropriate range, the trained model may experience overfitting.

**Table 7:** The prediction errors of different training epochs. (Hidden neurons = 200, hidden layer = 1).

Index	Hidden layers	Hidden neurons	Epochs	RMSE	MAE	MAPE	R^2^
DJIA	1	200	50	139.925	96.370	0.006	0.951
	1	200	100	133.657	95.981	0.006	0.955
	1	200	150	125.381	85.289	0.005	0.961
	1	200	200	126.100	96.809	0.006	0.960
	1	200	250	198.823	171.140	0.010	0.901
	1	200	300	**110.210**	**81.113**	**0.005**	**0.970**
S&P500	1	200	50	16.926	11.656	0.006	0.943
	1	200	100	**14.523**	**10.379**	**0.005**	**0.958**
	1	200	150	16.250	11.094	0.005	0.948
	1	200	200	19.882	15.717	0.008	0.922
	1	200	250	15.240	11.128	0.005	0.954
	1	200	300	14.824	11.652	0.006	0.957
HangSeng	1	200	50	310.126	237.797	0.010	0.984
	1	200	100	215.227	144.319	0.006	0.992
	1	200	150	176.751	121.313	0.005	0.995
	1	200	200	172.786	120.997	0.005	0.995
	1	200	250	158.074	111.722	0.005	0.996
	1	200	300	**138.132**	**92.056**	**0.004**	**0.997**
Nikkei225	1	200	50	616.210	465.037	0.026	0.847
	1	200	100	406.219	334.019	0.018	0.933
	1	200	150	868.720	679.180	0.038	0.696
	1	200	200	**236.780**	**178.897**	**0.010**	**0.977**
	1	200	250	509.599	411.324	0.022	0.895
	1	200	300	335.423	254.527	0.014	0.955
CSI300	1	200	50	**89.253**	**56.080**	**0.015**	**0.976**
	1	200	100	108.358	48.077	0.013	0.965
	1	200	150	119.622	47.654	0.013	0.957
	1	200	200	158.343	60.136	0.016	0.925
	1	200	250	167.441	49.244	0.014	0.916
	1	200	300	178.107	55.090	0.015	0.905
Nifty50	1	200	50	76.832	60.038	0.007	0.971
	1	200	100	64.743	50.256	0.006	0.979
	1	200	150	60.734	46.620	0.006	0.982
	1	200	200	56.663	43.503	0.005	0.984
	1	200	250	**52.686**	**39.986**	**0.005**	**0.986**
	1	200	300	78.060	67.690	0.008	0.970

#### 5.1.3 The impact of different numbers of hidden layers on experimental results

Finally, the effect of using different numbers of hidden layers is considered. It can be seen Table 8 that increasing the number of LSTM layers will affect the results. Adding LSTM layers helps improve the ability of neural networks to extract data features. However, in regard to different datasets, the optimal model parameters can only be obtained by selecting an appropriate number of layers for experiments.

**Table 8:** The prediction errors of using different number of hidden layers. (Hidden neurons = 200, epochs = 200).

Index	Hidden layers	Hidden neurons	Epochs	RMSE	MAE	MAPE	R2
DJIA	1	200	200	**126.100**	**96.809**	**0.006**	**0.960**
	2	200	200	192.442	163.038	0.009	0.907
	3	200	200	287.778	260.480	0.015	0.793
S&P500	1	200	200	**19.882**	**15.717**	**0.008**	**0.922**
	2	200	200	66.722	64.637	0.031	0.119
	3	200	200	28.687	23.942	0.012	0.837
HangSeng	1	200	200	**172.786**	**120.997**	**0.005**	**0.995**
	2	200	200	196.162	137.586	0.006	0.993
	3	200	200	275.657	220.294	0.010	0.987
Nikkei225	1	200	200	236.780	178.897	0.010	0.977
	2	200	200	**231.176**	**191.873**	**0.011**	**0.978**
	3	200	200	359.016	300.790	0.016	0.948
CSI300	1	200	200	**158.343**	**60.136**	**0.016**	**0.925**
	2	200	200	215.062	64.767	0.018	0.861
	3	200	200	187.716	78.538	0.020	0.894
Nifty50	1	200	200	**56.663**	**43.503**	**0.005**	**0.984**
	2	200	200	225.719	212.761	0.026	0.747
	3	200	200	440.930	414.905	0.050	0.034

#### 5.1.4 PSO-LSTM model with optimal parameters

**Fig 6:**
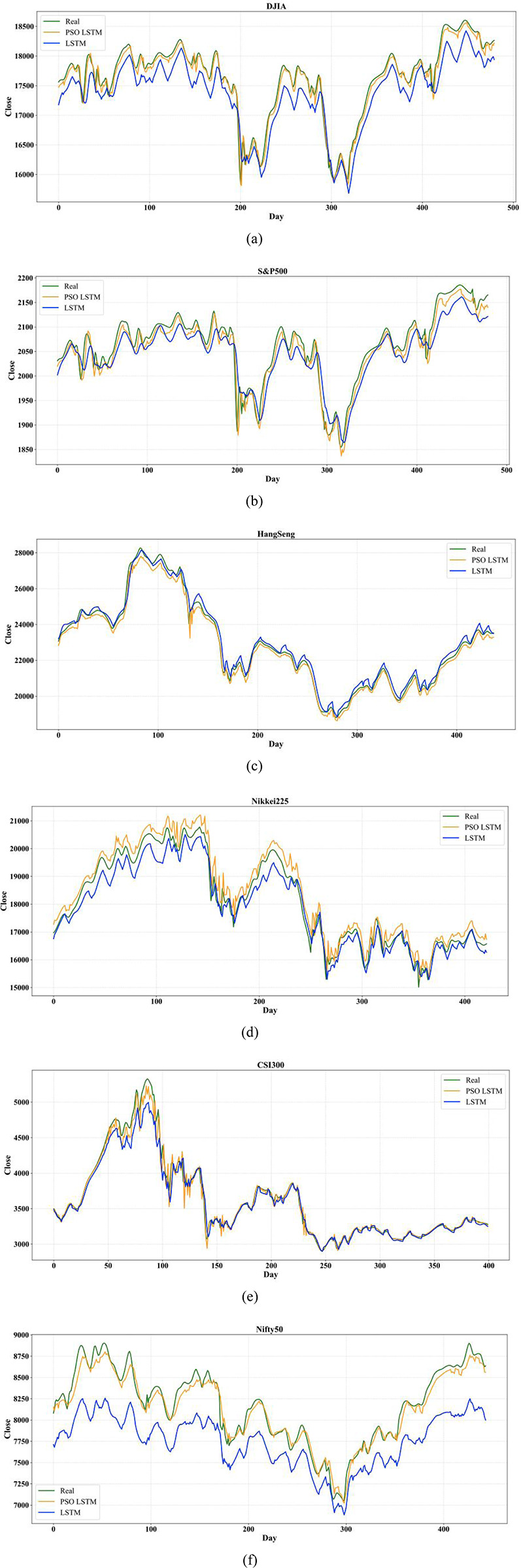
The line charts of real and predicted close price in 6 indices: (a) DJIA, (a) S&P500, (c) HangSeng, (d) Nikkei225, (e) CSI300, and (f) Nifty 50.

The prediction errors of the optimal parameters of the LSTM model are summarized in the Table 9 for the six stock indices representing different development markets. The comparison between the true and predicted values of the experimental dataset using LSTM and PSO-LSTM models are shown in Fig 6, respectively.

**Table 9:** The prediction errors of the optimal parameters.

Index	Hidden layers	Hidden neurons	Epochs	RMSE	MAE
DJIA	1/200/300	**110.210**	**81.113**	**0.005**	**0.970**
	2/200/200	192.442	163.038	0.009	0.907
	3/200/200	287.778	260.480	0.015	0.793
S&P500	1/200/100	**14.523**	**10.379**	**0.005**	**0.958**
	2/200/200	66.722	64.637	0.031	0.119
	3/200/200	28.687	23.942	0.012	0.837
HangSeng	1/200/300	**138.132**	**92.056**	**0.004**	**0.997**
	2/200/200	196.162	137.586	0.006	0.993
	3/200/200	275.657	220.294	0.010	0.987
Nikkei225	1/50/100	312.286	239.183	0.013	0.961
	2/200/200	**231.176**	**191.873**	**0.011**	**0.978**
	3/200/200	359.016	300.790	0.016	0.948
CSI300	1/200/50	**128.263**	**59.518**	**0.016**	**0.951**
	2/200/200	215.062	64.767	0.018	0.861
	3/200/200	187.716	78.538	0.020	0.894
Nifty50	1/50/100	**69.764**	**53.587**	**0.007**	**0.976**
	2/200/200	225.719	212.761	0.026	0.747
	3/200/200	440.930	414.905	0.050	0.034

### 5.2 Experiments of PSO-LSTM model (PSO automatically optimize parameters)

**Fig 7:**
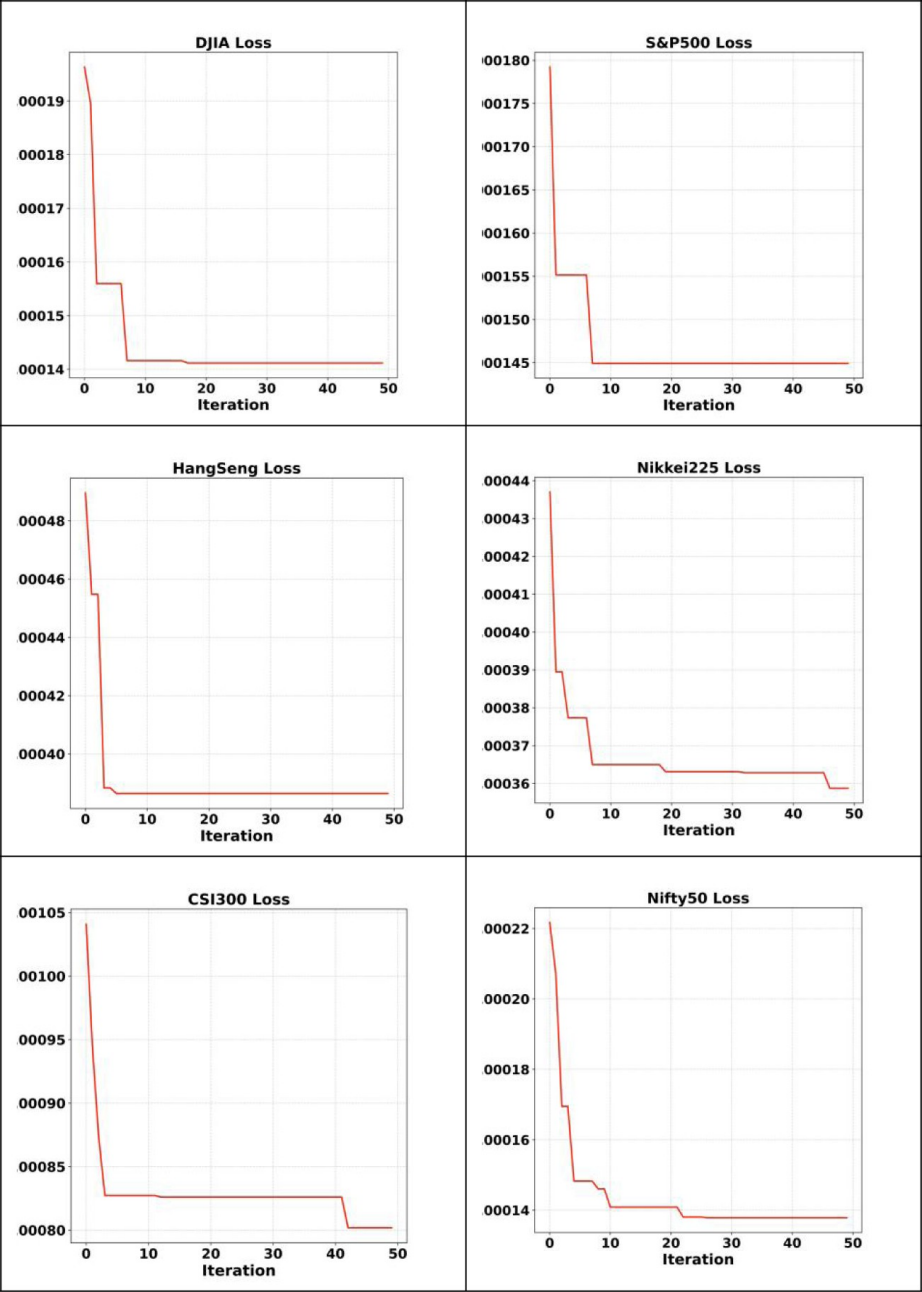
Loss change in iteration of 6 stock indices.

In section 5.1, experiments with manually setting parameters to determine the appropriate neural network model for prediction are conducted. In section 5.2, the hyperparameters of the LSTM neural network are automatically optimized by PSO. In Section 5.2, a 50 day retrospective period is used to validate the model. The accuracy of the neural network before and after optimization will be compared. The data from six stock indices proves the degree of fit between the predicted values of the PSO-LSTM model and the actual values. The prediction errors and optimal parameters of LSTM and PSO-LSTM models are listed in Table 10. The changes in fitness (MSE) of the PSO-LSTM model during the evolution process are shown in Fig 7. As the number of iterations of the PSO algorithm increases, the loss gradually decreases. The PSO-LSTM model performs better than the LSTM model in almost all different network layers and experimental data. Hyperparameter alternatives of the PSO-LSTM model are seen in Table 10. PSO and search and try to find best hyperparameters. The settings of algorithm parameter values are shown in Table 11.

**Table 10:** Hyperparameter alternatives.

Hyperparameter	Alternatives
Number of neurons	50,51,. . .,300
Hidden layers	1,2,3
Layer exist or not exist	0,1
Training epochs	50,51,. . .,300

**Table 11:** PSO parameter values.

parameter	value
sequence length	50
train_test_split	0.80
batch_size	32
loss	MSE
optimizer	Adam
N	20
D	6
K	50
w	0.8
c1	1.5
c2	1.5

**Fig 8:**
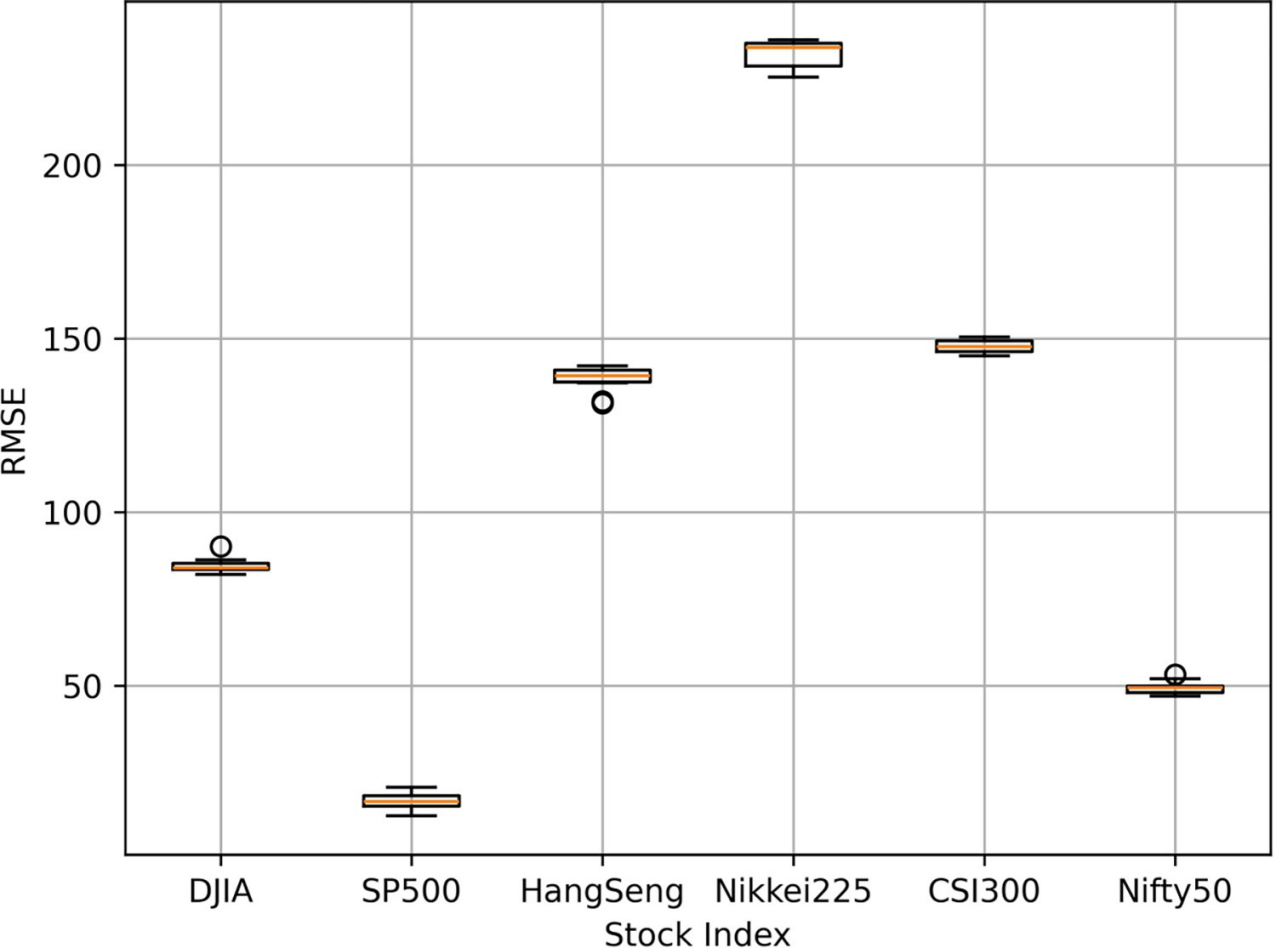
Box plots of the performance scores of PSO-LSTM model with 10 replications.

As mentioned above, PSO is used to optimize LSTM’s parameters. To validate the effectiveness, the comparison of LSTM parameters and errors before and after using PSO algorithm is expressed in Table 12. Furthermore, Fig 8 shows the box plots of the performance scores of PSO-LSTM model with 10 replications. Table 13 illustrates the performance scores of the PSO-LSTM models in the test data.

**Table 12:** Comparison of LSTM parameters and errors before and after using PSO algorithm.

Index	Methods	RMSE	MAE	MAPE	R^2^	Parameters
DJIA	LSTM	110.210	81.113	0.005	0.970	1/200/300
	**PSO-LSTM**	**87.261**	**58.813**	**0.338**	**0.981**	**1/258/300**
	LSTM	192.442	163.038	0.009	0.907	2/200/200
	**PSO-LSTM**	**83.381**	**55.117**	**0.317**	**0.983**	**2/156/296**
	LSTM	287.778	260.480	0.015	0.793	3/200/200
	**PSO-LSTM**	**87.965**	**70.084**	**0.400**	**0.981**	**3/234/286**
S&P500	LSTM	14.523	10.379	0.005	0.958	1/200/100
	PSO-LSTM	16.622	12.240	0.597	0.945	1/168/256
	LSTM	66.722	64.637	0.031	0.119	2/200/200
	PSO-LSTM	27.355	24.892	1.210	0.852	2/198/222
	LSTM	28.687	23.942	0.012	0.837	3/200/200
	PSO-LSTM	**16.196**	**13.359**	**0.650**	**0.948**	**3/130/246**
HangSeng	LSTM	**138.132**	**92.056**	**0.004**	**0.997**	**1/200/300**
	PSO-LSTM	151.459	102.642	0.443	0.996	1/178/284
	LSTM	196.162	137.586	0.006	0.993	2/200/200
	PSO-LSTM	151.282	101.889	0.441	0.996	2/98/236
	LSTM	275.657	220.294	0.010	0.987	3/200/200
	PSO-LSTM	267.336	221.079	0.939	0.988	3/237/300
Nikkei225	LSTM	312.286	239.183	0.013	0.961	1/50/100
	PSO-LSTM	**230.675**	**168.543**	**0.945**	**0.979**	**1/300/300**
	LSTM	231.176	191.873	0.011	0.978	2/200/200
	PSO-LSTM	231.192	169.667	0.947	0.978	2/198/285
	LSTM	359.016	300.790	0.016	0.948	3/200/200
	PSO-LSTM	231.192	169.667	0.947	0.978	3/262/186
CSI300	LSTM	128.263	59.518	0.016	0.951	1/200/50
	PSO-LSTM	166.910	61.631	1.620	0.916	1/232/258
	LSTM	215.062	64.767	0.018	0.861	2/200/200
	PSO-LSTM	**148.248**	**59.923**	**1.576**	**0.934**	**2/255/240**
	LSTM	187.716	78.538	0.020	0.894	3/200/200
	PSO-LSTM	153.872	53.093	1.402	0.929	3/202/288
Nifty50	LSTM	69.764	53.587	0.007	0.976	1/50/100
	PSO-LSTM	82.799	72.084	0.876	0.966	1/288/300
	LSTM	225.719	212.761	0.026	0.747	2/200/200
	PSO-LSTM	**49.931**	**38.210**	**0.464**	**0.988**	**2/156/222**
	LSTM	440.930	414.905	0.050	0.034	3/200/200
	PSO-LSTM	173.265	162.134	1.956	0.851	3/234/267

**Table 13:** The performance scores of the PSO-LSTM models in the test data.

Metrics		DJIA	SP500	HangSeng	Nikkei225	CSI300	Nifty50
RMSE	Min	80.689	12.818	132.232	226.678	144.970	46.401
	Avg	83.676	16.170	135.560	230.387	148.989	50.725
	Max	89.874	19.028	138.406	234.197	151.893	53.743
	Std	6.124	5.800	3.221	4.760	4.629	5.439
MAPE	Min	0.260	0.458	0.289	0.868	1.339	0.871
	Avg	0.366	0.636	0.915	1.016	1.561	1.908
	Max	0.546	0.776	1.411	1.202	1.857	3.438
	Std	0.008	0.010	0.103	0.014	0.030	0.391
R^2^	Min	0.949	0.926	0.990	0.967	0.924	0.974
	Avg	0.978	0.946	0.996	0.981	0.938	0.988
	Max	0.991	0.961	0.998	0.991	0.951	0.993
	Std	0.001	0.001	0.000	0.001	0.000	0.000

### 5.3 Experiments of other well known forecasting methods

#### 5.3.1 Comparison of machine learning model

There are many other classic algorithms for time series prediction. In this section, PSO-LSTM model proposed in this article will be compared with several popular methods adopted by researchers in recent years. In addition, the performance of the model under different prediction cycles can be more comprehensively evaluated by conducting experiments on retrospective periods of different time spans. In Section 5.3, a 50 day retrospective period is used to validate the model. In section 5.4, a 20-day and 7-day lookback period will be used to validate the model.

XGBoost can parallelly combine multiple weak classifiers (decision trees) into a strong classifier (elevation tree) through result weighting, making it one of the most efficient and high-performance algorithms in the engineering field [50]. XGBoost is often combined with deep learning models [48] or intelligent optimization algorithms [51, 52] for time series prediction. RF is an ensemble classifier based on Bagging proposed by Breiman [53]. RF uses the best classification result from all decision trees as the final result, which has been widely used in the field of stock prediction [54]. K-Nearest Neighbor (KNN) neural network algorithm lies in an accurate classification method, which classifies adjacent data parameters [55]. KNN has been successfully applied in financial time series forecasting [56]. Support Vector Machine (SVM) is a machine learning algorithm [57]. SVM is effective for regression and classification problems with multivariate features, which is also applicable to multivariate data for stock price prediction. The performance of support vector machines depends on the selection of kernel functions. Therefore, in practical problems, it is very important to choose a suitable kernel function to establish an SVM algorithm based on real data models. Support vector machines have been widely used in time series prediction [58–60]. Multi-layer perceptron (MLP) is a feed forward artificial neural network model. MLP neural network simplifies the structure of biological neurons to obtain the basic structure of the neural network, which is widely used for time series prediction [61, 62]. Bi directional long short-term memory (Bi-LSTM) model is a method of continuous input information based on LSTM with two input sequences, positive and negative. It is a special variant of recurrent neural networks, while retaining the advantages of LSTM in processing long-term correlated sequences and compensating for the disadvantage of LSTM in not using contextual information for prediction. This method has achieved good results in interactive prediction [63–65].

As for SVM, different activation functions are tested and the best results are obtained through RBF activation functions. Therefore, the parameter Gamma of RBF kernel needs to be adjusted. The optimal performance of SVM was *gamma* = 0.01. As for MLP, there is no fixed rule for selecting the number of hidden layers and the optimal number of neurons in the hidden layers. In order to compare the performance of the MLP and PSO-LSTM, the number of neurons, number of hidden layers, learning rate, number of training rounds, and other parameters of MLP were configured to be similar to PSO-LSTM. As for KNN, the best value of *k* was found to be 5 through testing, and the best values for different datasets were listed in the table. In all experiments, the first 80% of the data were used as the training set, and the last 20% were used as the testing set. This process was repeated 10 times. Finally, based on all experimental results, the average values are recorded as the final results. This technique is used to estimate the accuracy of prediction models in practical applications and avoid overfitting problems.

From the experimental results of the six datasets listed in Table 14, it can be seen that PSO-LSTM achieved the best performance in the HangSeng, Nikkei225, and Nifty50 datasets. PSO-LSTM ranks in the top three in predictive performance on DJIA, S&P500, and CSI300 datasets. Compared with seven other machine learning models, PSO-LSTM achieved excellent predictive performances.

**Table 14:** Predicting errors of 50 days look back of different methods.

	Method	RMSE	MAE	MAPE	R^2^
DJIA	XGB	668.906	560.761	3.122	-0.119
	RF	458.716	352.101	1.956	0.474
	KNN	652.675	547.290	3.050	-0.065
	SVM	**71.051**	**51.025**	**0.293**	**0.987**
	MLP	222.073	165.908	0.936	0.877
	LSTM	186.956	154.680	0.882	0.913
	BiLSTM	163.654	116.675	0.671	0.933
	RNN	752.102	695.160	3.896	0.415
	GRU	192.865	177.683	1.009	0.907
	PSO-LSTM	83.381	55.117	0.317	0.983
S&P500	XGB	80.587	64.844	3.078	-0.285
	RF	75.272	59.985	2.847	-0.121
	KNN	85.661	71.354	3.395	-0.451
	SVM	26.930	20.983	1.002	0.857
	MLP	**12.959**	**8.886**	**0.438**	**0.967**
	LSTM	29.190	26.037	1.265	0.831
	BiLSTM	99.303	96.890	4.677	0.950
	RNN	25.047	24.201	1.779	0.927
	GRU	18.082	15.593	0.757	0.935
	PSO-LSTM	16.196	13.359	0.650	0.948
Hangseng	XGB	907.446	395.809	1.499	0.860
	RF	873.127	374.359	1.414	0.871
	KNN	899.990	419.067	1.604	0.863
	SVM	574.173	249.319	0.951	0.944
	MLP	200.490	124.387	0.521	0.993
	LSTM	244.487	180.091	0.773	0.990
	BiLSTM	316.596	242.899	1.042	0.983
	RNN	1181.730	629.904	2.441	0.763
	GRU	145.441	98.938	0.428	0.996
	PSO-LSTM	**138.132**	**92.056**	**0.004**	**0.997**
Nikkei225	XGB	1444.590	1024.060	5.266	0.158
	RF	1380.340	966.803	4.961	0.232
	KNN	1387.305	988.759	5.084	0.224
	SVM	1058.519	664.636	3.370	0.548
	MLP	389.981	294.960	1.560	0.939
	LSTM	855.244	631.065	3.497	0.705
	BiLSTM	760.377	625.776	3.335	0.767
	RNN	833.963	657.412	3.435	0.720
	GRU	196.214	150.178	0.835	0.984
	PSO-LSTM	**230.675**	**168.543**	**0.945**	**0.979**
CSI300	XGB	455.732	220.223	4.883	0.375
	RF	443.674	208.939	4.599	0.408
	KNN	440.559	208.496	4.603	0.416
	SVM	432.689	165.925	3.543	0.437
	MLP	115.706	65.411	1.598	0.960
	LSTM	150.078	82.193	2.069	0.932
	BiLSTM	**96.917**	**59.633**	**1.595**	**0.972**
	RNN	142.482	74.807	1.784	0.939
	GRU	152.486	54.686	1.503	0.930
	PSO-LSTM	148.248	59.923	1.576	0.934
Nifty50	XGB	130.121	89.565	1.069	0.916
	RF	106.406	70.506	0.842	0.944
	KNN	148.732	101.055	1.215	0.890
	SVM	82.639	51.018	0.609	0.966
	MLP	143.099	111.152	1.354	0.898
	LSTM	130.354	112.966	1.358	0.916
	BiLSTM	575.555	551.918	6.670	0.646
	RNN	550.528	501.419	6.081	-0.506
	GRU	54.517	41.718	0.508	0.985
	PSO-LSTM	**49.931**	**38.210**	**0.464**	**0.988**

The p-value of the symbol test for the algorithm proposed in this article is 0.0078125 compared to other machine learning algorithms. The p-value of the Wilcoxon rank test is 0.005411. The difference between the PSO-LSTM algorithm proposed in this article and other traditional methods is at the level of 0.05 (95%), demonstrating the significant advantage of the proposed algorithm.

#### 5.3.2 Comparison of state-of-the-art models

The comparative experimental results between PSO-LSTM and other state-of-the-art (SOTA) models are shown in Table 15. The comparative experiment includes two situations: using different datasets and using the same dataset with different time periods. The main contribution of this article is to propose a model for optimizing the structure of neural networks.

**Table 15:** Comparison of SOTA models.

Model	Dataset	RMSE	MAE	MAPE	R2
WT-PSO-LSTM	S&P500	15.571	12.052	0.588	0.952
DMPSO-LSTM [66]	SZ	160.3716	118.796	0.081	0.971
CEEMD_CNN_LSTM [67]	CSI300	14.935	12.352	0.058	NA
CNN_GRU [68]	CSI500	75.766	58.014	NA	0.539

### 5.4 Experiments on different lookback periods (20 and 7 day lookback periods)

In sections 5.1 and 5.2, the experiments are conducted based on a 50 day retrospective period. In section 5.4, the performances under different lookback periods are further investigated. The changes at the current time point are predicted by analyzing the lookback period data of the S&P500 dataset. The results are listed in Table 14. It can be seen that there are no significant difference between using a 50 day (results of S&P500 of Table 14 in Section 5.3) and a seven day retrospective period (Table 16) and a twenty day retrospective period (Table 17) for prediction.

**Table 16:** Predicting errors of seven days look back in S&P500.

	Method	RMSE	MAE	MAPE	R2
S&P500	XGB	80.587	64.844	3.078	-0.285
	RF	75.398	60.108	2.853	-0.124
	KNN	85.661	71.354	3.395	-0.451
	SVR	26.930	20.983	1.002	0.857
	MLP	36.072	30.030	1.456	0.743
	LSTM	27.831	23.444	1.139	0.847
	BiLSTM	69.336	66.162	3.191	0.049
	RNN	34.641	28.255	1.370	0.763
	GRU	27.581	25.651	1.245	0.85
	PSO-LSTM	15.571	12.052	0.588	0.952

**Table 17:** Predicting errors of twenty days look back in S&P500.

	Method	RMSE	MAE	MAPE	R2
S&P500	XGB	80.587	64.844	3.078	-0.285
	RF	75.413	60.099	2.853	-0.125
	KNN	85.661	71.354	3.395	-0.451
	SVR	26.930	20.983	1.002	0.857
	MLP	45.726	32.712	1.580	0.586
	LSTM	22.931	18.699	0.910	0.896
	BiLSTM	60.627	57.539	2.778	0.273
	RNN	25.569	22.312	1.015	0.819
	GRU	16.294	13.758	0.670	0.947
	PSO-LSTM	16.262	12.278	0.601	0.948

As depicted in the table, the proposed model consistently outperforms alternative machine learning models across various retrospective periods (7 days, 20 days, and 50 days), with a minimum 25% increase in prediction accuracy. These findings underscore the robustness and applicability of the PSO-LSTM model, particularly in high-frequency trading scenarios.

## 6. Conclusion

The proposed PSO-LSTM model offers a novel approach to address the intricate challenge of stock price prediction. Although LSTM neural networks have gained prominence for their adeptness in handling financial time series data, their efficacy in practical scenarios is hampered by the intricate process of parameter optimization. This study introduces a hybrid model that integrates LSTM with PSO to mitigate this limitation and enhance predictive accuracy.

The PSO-LSTM model’s adaptability is particularly noteworthy, as it efficiently determines optimal parameters by leveraging the inherent problem-solving capabilities of the PSO algorithm. By swiftly identifying parameter combinations aligned with data characteristics, the model streamlines computational overhead and bolsters predictive performance. Empirical evaluations underscore the efficacy of the proposed approach. Manual exploration of key parameters, including the number of hidden neurons and layers, indicates their nuanced impact on model performance. Furthermore, automated optimization via PSO demonstrates tangible improvements in predictive accuracy as well as lower prediction errors.

Comparative analysis with traditional LSTM models and a spectrum of machine learning algorithms reaffirm the superiority of the PSO-LSTM framework. Notably, it emerges as a top-performer across diverse datasets, underscoring its robust predictive capabilities. Moreover, the model’s resilience is evidenced through retrospective validation across varying timeframes, wherein consistent predictive accuracy is maintained over 50, 20, and 7-day intervals. In essence, the proposed PSO-LSTM model represents a significant advancement in stock market prediction methodologies, offering a potent amalgamation of LSTM’s temporal modeling prowess and PSO’s optimization finesse.

The reasons that the model produces better results are as follows. Firstly, LSTM can capture the long short-term dependencies in financial time series, which can effectively learn and predict complex patterns and trends in data. Secondly, PSO algorithm is used to optimize the parameters of the LSTM model. PSO maintains a good balance between global search and local search to avoid the problem of falling into local optima. PSO also can find parameter combinations close to the global optimum in a short period of time. The advantages enable the PSO-LSTM model to be trained with the optimal parameter configuration to improve predictive performance. During the training process, various regularization techniques, such as dropout and L2 regularization, are employed to prevent overfitting of the model. Overfitting refers to the situation where a model performs well on training data but performs poorly on test data. The regularization process ensures the generalization ability of the model, making its predictions more accurate on unknown data. In addition, data normalization, denoising, and feature selection are conducted before model training. These steps ensure the quality of input data and enable the model to learn and capture important information of the data to improve prediction performance. Finally, a multi-level evaluations of the performances of the model are conducted. In addition, the performance of the model under different market conditions are also tested, which verify its adaptability and robustness in different scenarios.

However, the proposed solution in this article still has many aspects for improvements. The proposed algorithm focuses on stock prediction as a single objective optimization problem with the aim of enhancing accuracy. In practical applications, it is often essential to efficiently train the model in response to market changes. In addition, the neural network operates as a "black box" model that sacrifices the interpretability and understandability of the original features. How to utilize an automatic and effective feature extraction process to reduce the dimensionality of the feature space and extract transformed features to create a new low-dimensional space are promising.

In future work, data completeness by incorporating natural language analysis of financial news will be enhance. The latest models like GANs and pre-trained models for stock price prediction are also worth exploring. Additionally, the LSTM neural network will be refined by exploring additional parameters. Alternative evolutionary algorithms for hyperparameter optimization will also be investigated. Future values of the stocks can be predicted with low error. The trading system built by the proposed model can give successful buy/sell/keep suggestions.

## Supporting information

S1 File (ZIP)
